# Performance Analysis of Machine Learning and Deep Learning Architectures on Early Stroke Detection Using Carotid Artery Ultrasound Images

**DOI:** 10.3389/fnagi.2021.828214

**Published:** 2022-01-27

**Authors:** S. Latha, P. Muthu, Khin Wee Lai, Azira Khalil, Samiappan Dhanalakshmi

**Affiliations:** ^1^Department of Electronics and Communication Engineering, SRM Institute of Science and Technology, Chennai, India; ^2^Department of Biomedical Engineering, SRM Institute of Science and Technology, Chennai, India; ^3^Department of Biomedical Engineering, Faculty of Engineering, Universiti Malaya, Kuala Lumpur, Malaysia; ^4^Faculty of Science and Technology, Universiti Sains Islam Malaysia, Nilai, Malaysia

**Keywords:** carotid artery, ultrasound image, machine learning, deep learning, stroke

## Abstract

Atherosclerotic plaque deposit in the carotid artery is used as an early estimate to identify the presence of cardiovascular diseases. Ultrasound images of the carotid artery are used to provide the extent of stenosis by examining the intima-media thickness and plaque diameter. A total of 361 images were classified using machine learning and deep learning approaches to recognize whether the person is symptomatic or asymptomatic. CART decision tree, random forest, and logistic regression machine learning algorithms, convolutional neural network (CNN), Mobilenet, and Capsulenet deep learning algorithms were applied in 202 normal images and 159 images with carotid plaque. Random forest provided a competitive accuracy of 91.41% and Capsulenet transfer learning approach gave 96.7% accuracy in classifying the carotid artery ultrasound image database.

## Introduction

Every year, in India, 26% of people die due to cardiovascular diseases, stroke because of artery stenosis is 75%, and heart attack is 42%. In the United States, one of the 19 deaths is due to stroke (Farah, 2018). Risk factors that may lead to stroke are physical inactivity, being obese, heavy drinking, use of illegal drugs, family history having a stroke and other cardiovascular diseases, cholesterol, high blood pressure, diabetes, and smoking. Other factors with increased stroke risk are race-, sex-, age-, and hormones-related problems.

Stroke is the third prominent reason for death in many developed countries (Benjamin et al., 2019). The common cause of stroke is the formation of atherosclerotic plaque in the carotid artery that can grow large enough to block blood flow leading to stenosis or rupture causing clots in the artery. Progressive intimal accumulation of protein, lipid, and cholesterol makes medium- and large-sized arteries, causing atherosclerosis. Atherosclerosis may be existing in body parts, such as infernal aorta, coronary artery, superficial femoral artery, and the common carotid artery bifurcation region. Strain in the arterial wall causes variance in clinical, mechanical, and molecular levels in the artery. The plaque formation is compensated by artery enlargement with no changes in the lumen region, where blood flows.

The mapping of features to any one of the classes in a computer-assisted diagnostic system is called classification. Machine learning algorithms that are used for biomedical image classification are neural network, backpropagation, support vector machine (SVM), adaptive binary tree-based SVM, decision trees, such as linear regression, logistic regression, random forest, k-nearest neighbor (KNN), k-means, Boltzmann machine, mean shift clustering, Markov statistics nonparametric techniques, and fuzzy-based classification methods.

Stimulated by the function and structure of the brain, an artificial neural network (ANN) was developed. A subset of machine learning, called deep learning, performs classification tasks directly from the images. The accuracy of deep learning sometimes exceeds human performance. The model extracts all the necessary features by itself and performs the classification. Transfer learning is a kind of deep learning which uses the learnt knowledge from some other data and uses that for the application in hand. Some of the transfer learning algorithms are Alexnet, Mobilenet, Imagenet, Capsulenet, etc.

Carl Azzopardi et al. (2020) used a deep neural network (DNN) to delineate lumen-intima boundary (LIB) and media-adventitia boundary (MAB) with a fully automatic segmentation technique. For the network stochastic gradient descent optimization problem, a new objective function was formulated. The invariant intensity data input was given to the network with a bimodal synthesis of amplitude and phase congruency. The performance in MAB and LIB detection was 96.2 and 92.5%, respectively. The study was made with just 15 images in each stenosis category which is not a sufficient number for deep learning-based segmentation. Images from different sources were not considered for learning, missing generalizability (Azzopardi et al., 2020).

Roy-Cardinal et al. (2019) extracted noninvasive vascular ultrasound elastography (NIVE) and ultrasound features, such as homodyned-K (HK), Nakagami parametric maps, log-compressed images. The algorithm identified large lipid area, calcification, ruptured fibrous cap presence, differentiation of nonvulnerable and vulnerable plaques, and confirming symptomatic and asymptomatic patients using a random forest classifier. The study population was 91, and only 5 cases with fibrous caps were involved. A balanced dataset may give better classification performance. Based on elastography and B mode gray-level features, the AUC obtained was 0.90 (95% CI 0.80–0.92, *p* < 0.001). The area of calcification accuracy obtained was 0.95 (95% CI 0.94–0.96, *p* < 0.001), performed using the above features. Area under the curve variation for other tasks varied between 0.79 and 0.97 (Roy-Cardinal et al., 2019).

Loizou et al. (2017) studied the texture variability in the ultrasound video to identify the presence of vulnerable plaque. The videos were intensity normalized, denoised, IMT segmented, and texture feature learned to find systole and diastole states. The texture was visibly variable for diastolic and systolic states. More gray-scale average was recorded for systole compared to diastole. Plaque structures had variable textures in both the states. Systole and diastole features combined gave better results. Borders of type 1 plaque were not identified by this method. Acoustic shadowing was produced in type V plaque and was not recognizable. The state diagram was improper for 2% of cases (Loizou et al., 2017).

Lekadir et al. (2017) proposed a CNN classification model for the different plaque constituents. Lipid core, calcified tissues, and fibrous caps were detected with a correlation of 0.90 related to clinical results. Based on the patch batched technique, 56 images were converted into 90,000 patches for the process. SVM with predefined image features gave an accuracy of 78.5%. The testing time taken for classifying each image was 52 ± 13 ms, and changes in accuracy were reduced by 0.003 by changing the patches between 9 × 9, 11 × 11, 13 × 13, and 15 × 15 (Lekadir et al., 2017). Pazinato et al. (2016) used the features of neighboring pixels for carotid image classification. On a dataset with calcium, lipids, muscles, fibrous, and blood tissues texture, gradient, statistical, and local binary pattern (LBP) features were used. Pixel-based machine learning classification was carried out on the normalized image following multiscale description. The method was computationally complex and did not focus on any particular machine learning algorithm. The technique applied in ultrasound tissue engineering achieved a classification accuracy of 73%, and was statistically verified (Pazinato et al., 2016).

Gastounioti et al. (2015) explained the importance of kinematic features for plaque analysis for a computer-aided diagnosis (CAD). Fisher discriminant ratio-based feature selection and SVM-based classification were performed. Applying texture features gave 80% of accuracy and kinematic features recorded 88% of accuracy. The accuracy of this proposed CAD has still lots of scope for improvement. AUC, specificity, and sensitivity improved by 0.70, 0.83, and 0.67, respectively (Gastounioti et al., 2015). Vegas-Sánchez-Ferrero et al. (2014) defined a gamma mixture model (GMM) for the subsampled RF images, and their parameters are useful features to identify various plaque tissues. The method outperformed in terms of plaque echogenicity and characteristics. It achieved an accuracy of 95.16% for four-class classifications and 86.56% for three-class classification, which can still be improved (Vegas-Sánchez-Ferrero et al., 2014).

Saba et al. (2021) proposed a classification approach for carotid artery ultrasound images using four machine learning models, one deep learning model, and one transfer learning model. He used the scattering principle of the plaque, where the symptomatic ones are more scattered than the asymptomatic ones (Saba et al., 2021). He achieved stable results for the characterization and classification of the carotid artery ultrasound images.

Classification of the carotid artery images to identify the presence of plaque deposit is performed by machine learning algorithms, CART decision tree, random forest, and logistic regression. Convolutional neural network (CNN)-based deep learning classification and Mobilenet and Capsulenet transfer learning approaches are performed in the carotid artery image database. The performance of these classification methods is analyzed with the true values confirmed by three radiologists.

In this article, section 2 gives the methodology, section 3 describes the results and discussions, and section 4 concludes the article.

## Methodology

This section defines the approach involved in the classification of the carotid artery ultrasound images. Feature extraction and selection are done to obtain the appropriate features. The selected features are given as input to the machine learning classification algorithms, CART decision tree, random forest, and logistic regression. The images are given as input to the CNN, transfer learning algorithms, Mobilenet and Capsulenet. The classification performance measures are used to identify the efficiency of the algorithms.

**FIGURE 1:**
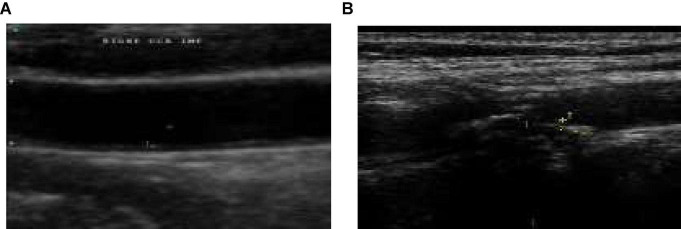
**(A)** Sample image without plaque deposit **(B)** with plaque deposit.

Figures 1A,B give the sample carotid artery ultrasound images with and without plaque deposit.

### Database Creation

Ethical clearance is obtained from the SRM Medical College Hospital and Research Center, Kattankulathur, Tamil Nadu, India, to collect carotid artery ultrasound images. Database of the carotid artery ultrasound B mode images is collected from the Bharat Scans, Chennai and the SRM Medical College Hospital and Research Center, Kattankulathur, Chennai.

### Feature Extraction

Machine learning involves high-dimensional data, where the analysis requires a considerable amount of data for learning and testing. The images obtained are denoised by curvelet decomposition to remove speckle and preserve useful edges. Feature reduction minimizes the effects of redundant variables by selecting feature subsets. Choosing the most significant features progresses the classification model performance and reduces over fitting.

Following preprocessing and segmentation of the images, 63 features are taken from the images in the database. A number of 33 texture features, 5 shape features, 10 histogram and correlogram features, and 15 morphology features are extracted from the images. Out of that, 22 most significant features are selected by principal component analysis (PCA) method (Parhizkar et al., 2021).

The most discriminant features from the extracted features are selected based on the following approach. Distance between two classes for every feature is computed as follows for mean m_1_, m_2_ and standard deviation σ_1_, σ_2_.


(1) $$\mathrm{distance}=\frac{\left|{\text{m}}_{1}-{\text{m}}_{2}\right|}{\sqrt{\sigma_{1}^{2}+\sigma_{2}^{2}}}$$

Features with more distance are those with more significance. From the 65 extracted features, 22 most significant features were selected for the classification task. PCA-based feature selection was performed in addition. The principal components are derived from the eigenvalues. A correlated feature set is converted into uncorrelated ones called principal components by an orthogonal transformation.

The selected features are texture, spatial structure, skewness, kurtosis, histogram, correlogram, histogram of oriented gradient (HOG), Gabor wavelet, angular 2nd moment, shape, sharpness, length irregularity, mean probability density function, gray-scale median, multiregion histogram, arterial wall ROI’s randomness, absolute gradient, radian and angular sum of discrete Fourier transform for Fourier power spectrum, coarseness, convexity, connectivity, and plaque volume. The potential features are given as input to the machine learning classification algorithms.

### Classification by Machine Learning Algorithms

Proper data preparation, automation and iterative learning, testing, scalability, and ensemble modeling are necessary for a classification algorithm. The classification of the carotid artery images database is performed with the machine learning algorithms, CART decision tree, logistic regression, and random forest algorithm.

Machine learning is to develop a mathematical model built by training the inputs. The inputs are the features selected from the ultrasound image dataset of the carotid artery. The learning experience is generalized so that it can give the correct output for the new image which is not in the database. The generalization of the model is improved by applying a validation set to the trained model. The resulting output and error are given as feedback to the input so that training of the model improves. After many iterations of tuning and training of the model, the trained model is used with new unseen test data to find the performance of the approach (Lundervold and Lundervold, 2019; Latha et al., 2020).

#### CART Decision Tree

The decision tree is a prediction-based machine learning model with parameters represented in the branches and target outputs represented in the form of leaves. Branch labels are represented by leaves and feature conjunctions that lead to the leaves are represented as branches. Target with continuous values is called regression trees. Classification and regression tree (CART) is a nonparametric decision tree algorithm (Seera and Lim, 2014). Information gain defines how to quantify the quality of the split. For attributes p and q, the information gain I is represented as


(2) $$\text{I}\,\left(p,q\right)=-\frac{\text{p}}{p+q}\,\log_{2}\left(\frac{\text{p}}{p+q}\right)-\frac{\text{q}}{p+q}\,\log_{2}\left(\frac{\text{q}}{p+q}\right)$$

To create a tree from the available attributes, entropy is computed. It depends on how much variance the data has.


(3) $$\text{E}\,\left(\text{A}\right)=\sum_{i=1}^{\text{v}}\frac{{\text{p}}_{\text{i}}+{\text{q}}_{\text{i}}}{p+q}\,I\,\left(p,q\right)$$

The training sets each attribute that is found from the gain. It is the variance between entropy and information gain.


(4) Gain=I⁢(p,q)-E⁢(A)

Decision trees can identify the nonlinearity in the dataset and adapt accordingly. The data need not be standardized because a distance measure is not involved in the classification. Sigmoid activation is used to get the optimum classification result. The rules of CART and other decision trees are as follows:

1. Based on a variable’s value, the splitting criteria for a node are formulated.
2. The stopping criteria are decided when to stop splitting a tree.
3. Final target variable at the end of each node is calculated.

**FIGURE 2:**
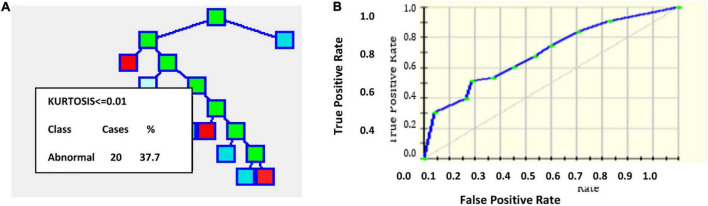
**(A)** Tree formation for sample 53 images with kurtosis feature **(B)** ROC curve.

An output of one implies the presence of plaque, and zero represents the absence of plaque in the image with a threshold of 0.5. Figure 2 gives the results of applying the CART decision tree for the carotid artery ultrasound image database. Using the kurtosis feature, the tree formation for sample 53 images is projected in Figure 2A. Kurtosis ≤ 0.01 is separated and branches are formed from that node. Figure 2B is the ROC curve for which the AUC is 83.53%, which implies that CART is suitable for disease classification in the carotid artery.

Classification and regression tree is nonparametric and hence is independent on the distribution kind of the input data. The algorithm is not affected by the outliers in the input data. Without strictly following the stopping rule, the tree can be overgrown and can be pruned back to the optimal solution. Fit can be improved using a test set and validation sets. The input variable set can be selected by combining CART with other prediction methods. The drawbacks of CART include variance in the model when a small change in the database is made and imbalanced class data lead to underfit trees.

#### Logistic Regression

Binary logistics is more suitable for categorical targets with linear or nonlinear decision boundaries, with a threshold fixed. It applies the logistic or sigmoid function. For the curve’s maximum value L, steepness parameter or growth rate k and x0 being the midpoint of x, the logistic function is given by


(5) $$f=\frac{\text{L}}{1+{\text{e}}^{-\text{k}\,\left(x-{\text{x}}_{0}\right)}}$$

Assuming threshold 0.5, for probability 0.5, class = 1 is assigned. For probability < 0.5, class = 0 is assigned (Barui et al., 2018). The cost function J used is crossentropy since sigmoid activation is used.


(6) $$\text{J}\left(\theta \right)=\frac{1}{\text{m}}\sum_{i=1}^{\text{m}}\mathrm{cost}({\text{h}}_{\theta }\left({\text{x}}^{\text{i}}\right),\left({\text{y}}^{\text{i}}\right)$$

Where cost(hθ(x),y) = −log(hθ(x)) for y = 1 and cost(h(x),y) = −log(1−h(x)) for y = 0. The natural log of odds called logit which transforms the line into the logistic curve is


(7) $$\log\left(\frac{\text{p}\,\left(\text{x}\right)}{1-p\,\left(\text{x}\right)}\right)=\beta_{0}+\beta_{1}\,\left(x\right)$$

**FIGURE 3:**
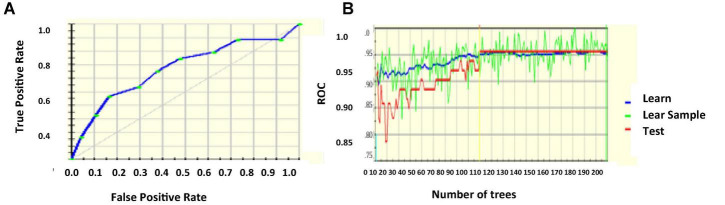
**(A)** ROC curve **(B)** number of trees with respect to ROC.

The logistic regression coefficients are found by maximum likelihood estimation. Highly correlated inputs from the database are removed after calculating the pair-wise correlation of the features. It is done to prevent overfit because of multiple highly correlated inputs. The sparsity of the data is also reduced so that the likelihood estimation does not prevent target convergence (Zhang et al., 2018; Javeed et al., 2019; Zhang and Han, 2020). Figures 3A,B project the ROC curve and the number of trees with AUC 87.55%.

#### Random Forest

Random forest is an ensemble classification approach, protecting the structure from being affected by overfitting problems, introduced by Ho in 1995. The tree learners of the random forest follow bootstrap aggregation bagging. Without increasing, bias bootstrapping reduces the variance of the model. The trees are uncorrelated so the prediction of the average of many trees is not noise-sensitive. Bootstrapping gives different input sets for each training time. A forest is created randomly with root, internal, and terminal nodes. Algorithm efficiency improves for a bigger tree. Unlike other decision tree algorithms, random forest decides the root and other nodes randomly.

The classifier is efficient enough to handle missing values and is more suitable for categorical classification. Random forest is created first, and predictions are made from the created forest (Javeed et al., 2019; Wu et al., 2020). Sigmoid activation function is used. Using the random nodes, incorrect labeling can be identified using Gini impurity given by


(8) $${\text{I}}_{\text{G}}\,\left(\text{n}\right)=1-\sum_{i=1}^{\text{j}}{\left({\text{p}}_{\text{i}}\right)}^{2}$$

The algorithm for random forest creation is as follows.

1. From a total of m feature sets, K features are randomly selected k < m.
2. Find node from features after best split point.
3. From the best divided, segregate child node.
4. The above steps are repeated until l number of nodes is achieved.
5. Repeat the above steps for *n* times to achieve *n* nodes.

The prediction that forms the created random forest is done by the below procedure.

1. For each test feature, the rules of the model are applied to get the target.
2. For each predicted target, the votes are estimated.
3. The more voted target is considered the outcome.

**FIGURE 4:**
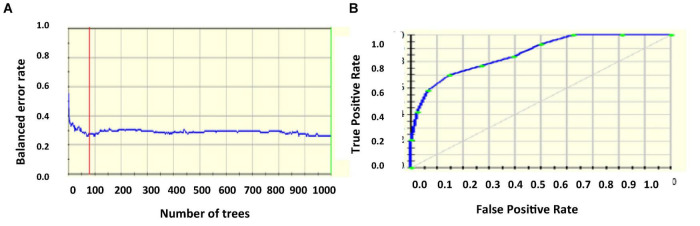
**(A)** Error rate **(B)** ROC curve.

Figure 4A projects the error rate which is least for nearly 85 number of trees, then increases, becomes constant, and the next drop is marked in nearly 920 trees. Figure 4B gives the ROC curve with AUC 90.63%.

Random forest combines individual tree’s decisions and considers the maximum voted one, which makes it one of the best machine learning algorithms. Trees are modeled more diversely, thus implementing all possible models, and obtaining all possible outcomes improves model efficiency. Kernel-induced random forest (KIRF) is followed where trees are built till error no longer reduces. Out of bootstrap (OOB) samples are applied to get the error rate of the random forest by taking the mean of the error from all the bags using all the available features. The drawbacks of the random forest include model complexity, more time consuming than other decision trees, and less intuitive for large decision trees.

### Deep Learning Algorithms

Deep learning, which is a class of ANN, extracts the semantic from the images directly, resulting in better classification performance. The deep learning model is built with multisource labeled data and provides more generalized results. The carotid artery ultrasound image classification is performed with a deep learning approach, CNN.

Deep learning is a promising machine learning field that can unravel artificial intelligence problems efficiently. It uses a DNN where the solution depends on the database. Deep learning is superior in terms of nonlinearity, generalization, harmony, fault tolerance, parallelism, and learning. There are undisclosed neural network layers that perform the learning for the available data. Each layer holds a relationship with the next and the previous layers. Deep learning absorbs features and useful representations directly from the raw image bypassing the feature extraction step. This automatic learning of feature representation and learning both happen in the layers.

Due to complexity, the importance of the subject, carotid image analysis using machine learning is not efficient enough and needs a model learnt from a huge number of images. The analysis does not depend on the features extracted manually. The data may be patient-dependent and expert-dependent which may influence the outcomes. Deep learning extracts the hidden feature representations of the images and helps in efficient diagnosis. For example, deep learning algorithms are CNN, DNN, DBM, LSTM networks, and generative adversarial networks (GANs), each having their pros and cons which does not require any preprocessing of data. The extension of CNN called transfer learning algorithms, such as Alexnet, Leenet, Googlenet, and Resnet, has proved their efficiency in the testing phase to a huge extent in terms of complexity.

Deep learning stacks many neuron layers constructing a hierarchical feature representation. The layer count in the model is over 1,000 creating a gigantic model memorizing all features and thus makes more intelligent classification.

Deep learning executes feature engineering on its own by combining and correlating the necessary attributes of the image. Deep learning solves the classification problem end-to-end, which makes the model better than other machine learning approaches. There is a lot of scope of development of deep learning with emerging techniques, such as transfer learning. Other challenges of deep learning are interpretability, trust, data, regulations, and workflow integration.

#### Convolutional Neural Network

Convolutional neural network is a proven traditional deep learning network based on its translation invariance property and shared weights architecture. All nodes connected to all nodes in the other layers build a much complex system and may be inefficient. CNN uses the domain knowledge of the data preserving the spatial relationship, assembling complex patterns into small, simple patterns (Tajbakhsh et al., 2016).

**FIGURE 5:**
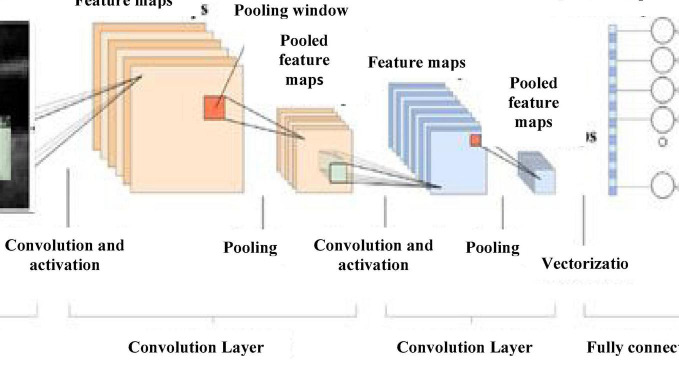
CNN architecture.

Rectified linear unit (ReLU) activation function is used for CNN activation. In convolution layer activation, previous layer activations are convolved with parameterized filters of size 3 × 3. Learning the same weight reduces the complexity of weight calculation for each layer and node. The convolution layer outputs are polled in a pooling layer. For small grids, the polling layer provides single output by max-pooling or average pooling. Translational invariance is achieved after the pooling layer preventing a shift in activation maps because of the shift in the input. Increased stride length convolution leads to downsampled pooling reducing the model complexity. Based on a stochastic sampling of the neural network, dropout regularization is performed. Different neurons are removed in different iterations leading to different outputs each time. Weights are updated each time to get more optimal results. Activation maps subtracted from the mean and divided by standard deviations for each training batch give batch normalized output (Lundervold and Lundervold, 2019). Figure 5 gives CNN architecture. The image is directly fed as input to the model. The convolution layer extracts features, such as corners, edges, and colors from the input image. Deeper layers extract more deep features, such as plaque structure, kurtosis, texture of plaque, and nonplaque area. Dominant features from the restricted neighborhood are extracted in the pooling layer.

Max-pooling representation is used, which minimizes computational cost and provides translational in-variation to the internal representation. Alternate convolution and pooling layers are used to reduce the large feature space. Later, layers extract more disease-related features assisting the classification process and improve classification accuracy.

After the convolution and pooling, the data are converted into a column vector, suitable for multilevel fully connected architecture. It is followed by a feed-forward neural network and back-propagation architecture in successive training iterations. Dominant and low-level features are adequately identified and classification proceeds.

#### Transfer Learning Based on Mobile Network Architecture

A network pretrained on available images can be fine-tuned for the application to be performed. When the source and the target are nearly similar, transfer learning works best in terms of weight updating and optimization compared to random initializations.

**FIGURE 6:**
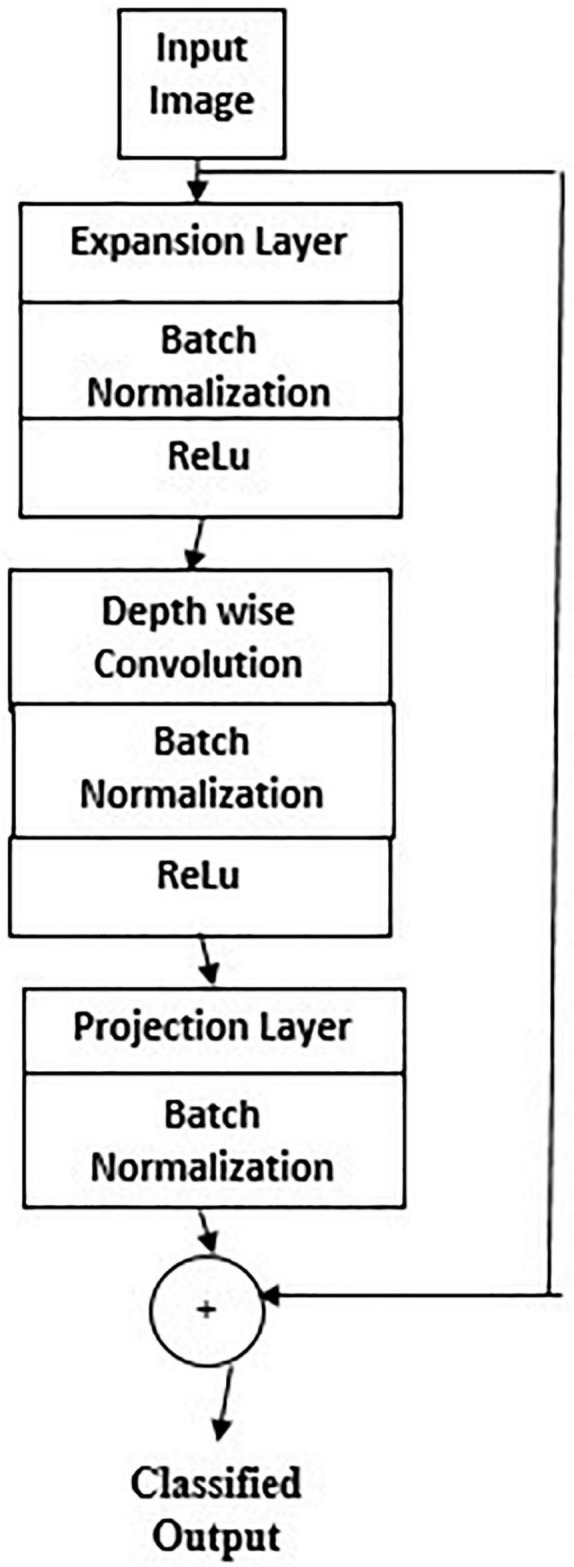
Mobilenet architecture.

Figure 6 gives Mobilenet architecture. The types of transfer learning are positive, negative, and neutral. Learning in a condition facilitating another condition is called positive transfer learning. Learning a task that makes learning another task harder is called negative learning. A learning which does not make a change in another learning is called neutral type of learning. A 1 × 1 convolution is associated with the depthwise convolution outputs in a pointwise convolution layer. In a single step, inputs and outputs are combined using a convolution filter. Using Mobilenet, computation and model size have drastically reduced. Transfer learning marks fast training, more accurate, and needs fewer data. The significant levels of transfer learning are

1. Full network adaptation—weights are updated from a pretrained network instead of arbitrary initialization and apprise them during the training phase (Wang et al., 2016).
2. Partial network adaptation—network parameters from the pretrained network are initialized and used as such for the first few layers and the last layers are updated for training (Zeng et al., 2017; Hesamian et al., 2019).
3. Zero adaptation—network parameters from a pretrained network are used and are not changed throughout.

Zero adaptation may not be suitable for medical images trained with other organs or general images because they may not have similar properties of the carotid image. In using this carotid database for testing a pretrained network, since the available dataset is small than the training dataset, the following procedure is followed. Overfitting may be a concern because of the small testing set (Akbarian et al., 2019; Latha et al., 2021). The extracted high-level features may not be similar to the target dataset. The key features of Mobilenet model compared with the CNN model are the following.

1. Most of the pretrained layers near the start of CNN are removed.
2. Instead, fully trained networks equal to the number of classes for the application are included.
3. The newly obtained weights are randomized and replaced instead of the removed network weights.
4. The network is trained to update the weights of the new fully connected layers.

Mobilenet is a family of mobile-first computer vision model for TensorFlow considering restricted data available and suited for embedded applications. The model is small, low latent, and low power designed by google researchers. A width multiplier parameter is introduced to overcome the resource-accuracy tradeoff. The resolution multiplier term reduces the layers’ internal structure. ReLU activation function is used.

**FIGURE 7:**
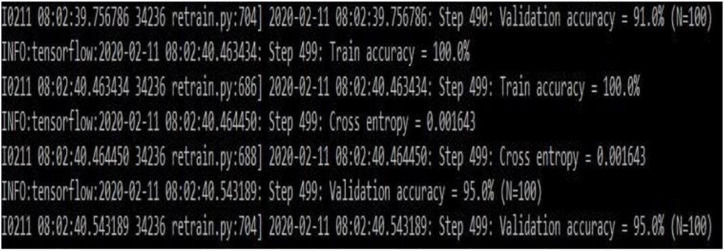
Transfer learning based on the Mobilenet architecture (snapshot of the obtained results).

Figure 7 gives the transfer learning with mobile net architecture, which provides training accuracy 100% and validation accuracy 95%. Though the training performance is less than that of CNN, the validation performance has improved drastically on using mobile net architecture.

#### Capsulenet

Geoffrey Hinton proposed Capsulenet in 2017, which is a better representation of capsules than convolution. The neuron activities also have a viewpoint variance in addition. CNN requires augmentation and depends more on texture features, which led to these transfer learning approaches. CNN’s max-pooling may lose valuable information because of poor relationships between hierarchies of simple and complex objects. Capsulenet applies vector activation and outputs which encodes feature transformation information. ReLU activation function is used.

**FIGURE 8:**
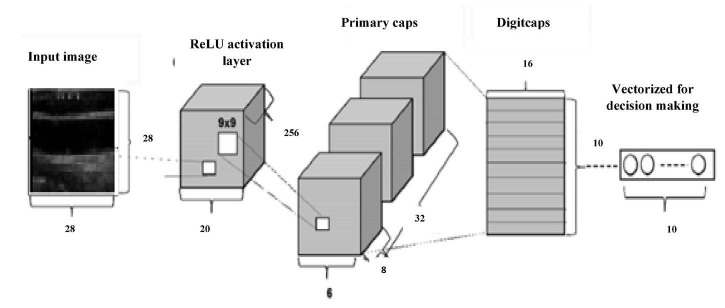
Capsulenet architecture.

Figure 8 gives Capsulenet architecture with ReLU activation. Capsules are convolutions with block nonlinearity and routing. The iterations are slow but require few parameters than CNN. Inside the knowledge representations, Capsulenet builds a better model hierarchy. Capsule structures are added to the CNN model, and the outputs are reused to get more stable higher representations. Max-pooling is used instead of dynamic routing and hence achieves translation invariance. It improves the ability of the network to detect an object even wherever it lies in the image.

## Results and Discussion

Choice of performance measures to evaluate the machine learning algorithms gives hope for its practical use. An unsuitable incorrect measure will mislead to wrong results and a flawed model which is not suitable for the application. The available data are imbalanced, and thus, analyzing more number of metrics assists in proper model selection. It involves comparing the proposed model with an existing model or predicting the class label for a given image set.

### Performance Metrics

The classification of a carotid artery ultrasound image as symptomatic or asymptomatic is a binary classification problem. The performance depends on the count of correctly classified samples to their class (true positive (TP)), not belonging to the class, correctly classified as (true negative (TN)), samples misclassified to that class (false positive (FP)), and those that are misrecognized as belonging to that category (false negative (FN)) (Sokolova and Lapalme, 2009). The overall effectiveness of the model is given by


(9) $$\mathrm{accuracy}=\frac{\mathrm{TP}+\mathrm{TN}}{\mathrm{TP}+\mathrm{TN}+\mathrm{FP}+\mathrm{FN}}$$

The labels class agreement with positive labels in the algorithm is given by


(10) $$\mathrm{precision}=\frac{\text{TP}}{\mathrm{TP}+\mathrm{FP}}$$

Positive label identification efficiency is expressed by recall or sensitivity. The relevant data points are identified using. F score measures the relationship between the positive labeled data and that given in the classifier. Specificity explains how effective the model identifies a negative label. FPR is the false alarm probability and TPR is the recall parameter. The model’s ability to identify false classification is derived from the area under the ROC curve (AUC). An AUC rate 1 is expected for an ideal classification model. These measures signify the classification model performance.


(11) $$\mathrm{recall}=\frac{\text{TP}}{\mathrm{TP}+\mathrm{FN}}$$

(12) $$\mathrm{precision}=\frac{\text{TP}}{\mathrm{TP}+\mathrm{FP}}$$

(13) $$\text{F}\,\mathrm{score}=2\,\text{×}\,\frac{\mathrm{precision}\times \mathrm{recall}}{\mathrm{precision}+\mathrm{recall}}$$

(14) $$\mathrm{specificity}=\frac{\text{TN}}{\mathrm{TN}+\mathrm{FP}}$$

(15) $$\mathrm{AUC}=\left(\frac{1}{2}\right)\,\left(\frac{\text{TP}}{\mathrm{TP}+\mathrm{FN}}+\frac{\text{TN}}{\mathrm{TN}+\mathrm{FP}}\right)$$

(16) $$\mathrm{Accuracy}=\frac{\mathrm{TP}+\mathrm{TN}}{\mathrm{TP}+\mathrm{TN}+\mathrm{FP}+\mathrm{FN}}$$

ReLu activation function is used in the classification models.

### Machine Learning

Table 1 gives the confusion matrix of the machine learning algorithms applied in the dataset containing 361 images, out of which 159 are abnormal and 202 are those without any disease indications.

**TABLE 1:** Confusion matrix of machine learning algorithms.

	CART Decision tree		Logistic regression		Random forest	
	Actual positive (1)	Actual negative (0)	Actual positive (1)	Actual negative (0)	Actual positive (1)	Actual negative (0)
Predicted positive (1)	123	34	120	27	132	23
Predicted negative (0)	23	181	14	200	8	198

The CART model gives an accuracy of 84.21%, specificity 88.72%, sensitivity 78.34%, and precision of 84.25%. The results prove that the model is useful in identifying the negative cases better than the positive ones. Logistic regression records an accuracy of 88.64% for the carotid database. The obtained specificity is 93.46%, sensitivity is 81.63%, and precision is 89.55%. More number of features added to the logistic regression model will increase the variance in the odds and may lead to overfitting. This reduces the generalization of the model fit. Based on the chi-square test, Hosmer–Lemeshow goodness-of-fit measure can improve model performance. The algorithm that assumes the data is noise-free. Outliers from the training data must be removed to prevent misclassification. Random forest gives an accuracy of 91.41%, specificity 96.11%, sensitivity 85.16%, and precision of 94.29%. The above results prove that random forest is a more accurate classifier than logistic regression and CART decision tree for classifying the carotid artery ultrasound images.

### Deep Learning

**FIGURE 9:**
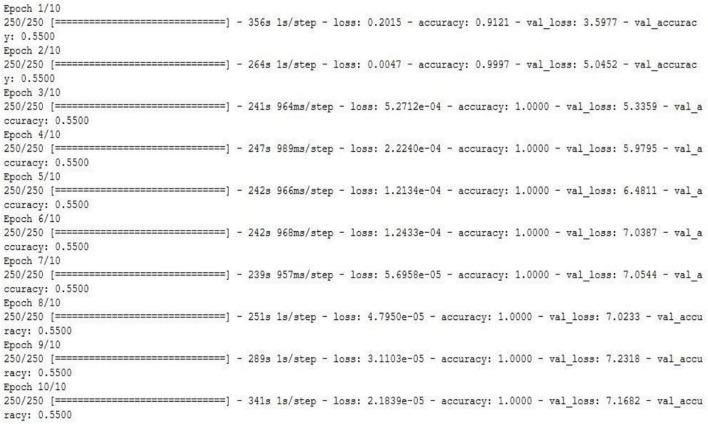
CNN model applied to the carotid artery ultrasound image database (snapshot of the obtained results).

**FIGURE 10:**
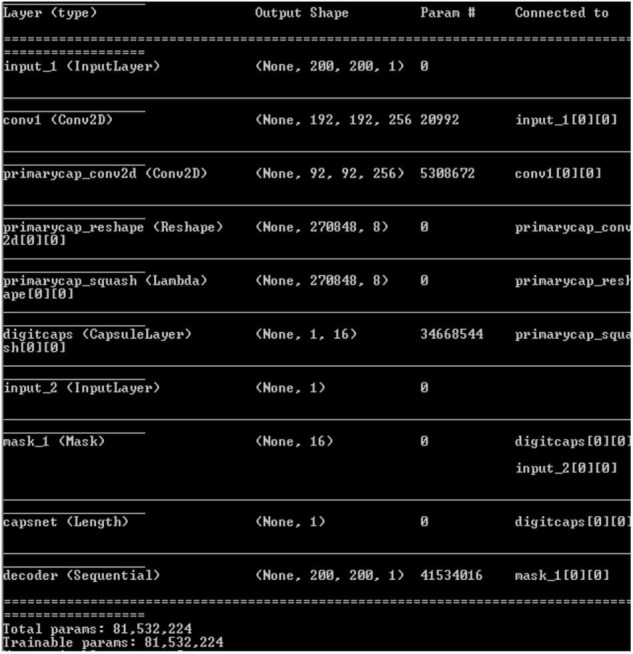
Capsulenet implementation for the carotid artery database images (snapshot of the obtained results).

Convolutional neural network model is applied on ultrasound image database for the classification of the images as with and without plaque deposit. The model achieved training accuracy of 100% and validation accuracy of 55% as given in Figure 9. Figure 10 gives the result of the capsulenet implementation in the database.

Convolutional neural network requires a wide number of data for training the model. Because of the limited number of data, the validation performance is nearly half, though the training is efficient. To overcome this, transfer learning was introduced to perform a deep learning architecture with limited training dataset.

Capsules group neurons and thus require fewer parameters between layers. Pose matrix in Capsulenet defines the rotation and translation of an object, which represents its change in viewpoint. It makes the model better generalized to new viewpoints. The spatial relationship between part of the image and the whole is learnt which makes the image identification simple. It is a viewpoint-dependent neural activity which does not require image normalization and can also identify multiply transformed images (Samiappan and Chakrapani, 2016; Arun et al., 2019; del Mar Vila et al., 2020; Samiappan et al., 2020). Underfitting problem was seen in the classification problem by CNN, which has led to poor performance and generalization. The carotid artery ultrasound image dataset is small and was not sufficient for a deep learning-based classification.

Initially, 300 training images and 61 validation images were used. Data augmentation methods, such as rotation, flipping, and translation were done to improve the classification accuracy.

Table 2 gives the performance of the three machine learning techniques applied for the carotid artery ultrasound image database. Random forest gives computationally faster and improved performance results compared to CART and logistic regression. Since the dataset was small (361 images), machine learning algorithms were not computationally complex, lags in accuracy of identification of the disease. Capsules group neurons and thus require fewer parameters between layers. Pose matrix captures rotated and translated versions as linear transformations, and so, Capsulenet is better generalized to new viewpoints. The spatial relationship between part of image and the whole is learnt, which makes the image identification simple. Capsulenet achieves accuracy of 96.7%, which is the highest for the carotid artery database images.

**TABLE 2:** Performance comparison of carotid artery image classification using machine learning approaches.

Algorithm	Accuracy (%)	Specificity (%)	Sensitivity (%)	Precision (%)	F score (%)	AUC (%)
CART Decision Tree	84.21	88.72	78.34	84.25	81.19	83.53
Logistic Regression	88.64	93.46	81.63	89.55	85.41	87.55
Random Forest	91.41	96.11	85.16	94.29	89.49	90.63

The images in the database were flipped to both plane axis rotated to π/4 axis. Table 3 gives the performance of the three deep learning techniques applied in the carotid artery image database.

**TABLE 3:** Performance comparison of carotid artery image classification by deep learning approaches.

Algorithm	Accuracy (%)
CNN	55
Mobilenet	95
**Capsulenet Transfer Learning**	96.7

Proposed Capsulenet with max-pooling gives 12.91, 8.33, 5.47, 43.12, and 1.75% improvement in accuracy compared with a CART decision tree, logistic regression, random forest, CNN, and Mobilenet classification algorithms, respectively. Negative transfer is the interference of the previous knowledge in the new learning. It has not affected the classification performance of the carotid artery ultrasound images. It is proved with improved performance measures.

It is proved that deep learning approaches give improved accuracy of 95.7% for Capsulenet compared to other machine learning and deep learning algorithms reported in the literature.

## Conclusion

A number of 361 images were processed to form a database with the help of radiologists. Extracted features from the database images are applied to the machine learning algorithms CART decision tree, random forest, logistic regression, CNN model, Mobilenet, and Capsulenet transfer learning algorithms for classifying the images as normal or abnormal. Machine learning algorithms were able to perform with an accuracy of 84.21, 88.64, and 91.41%, respectively, for CART, logistic regression, and random forest. Proposed Capsulenet transfer learning approach eliminates the need for large amount of training data. Proposed Capsulenet with max-pooling gives 12.91, 8.33, 5.47, 43.12, and 1.75% improvement in accuracy compared with CART decision tree, logistic regression, random forest, CNN, and Mobilenet classification algorithms, respectively.

## Data Availability Statement

The original contributions presented in the study are included in the article/supplementary material, further inquiries can be directed to the corresponding authors.

## Ethics Statement

Ethical clearances were obtained from SRM Medical College Hospital and Research Center, India. Ethics Clearance Number: 1736/IEC/2019. The patients/participants provided their written informed consent to participate in this study.

## Author Contributions

SL performed conceptualization, methodology, design, data collection, data visualization, formal analysis, reviewing, and editing. PM carried out conceptualization, methodology, design, investigation, data collection, data analysis, and writing original draft preparation. KL done conceptualization, methodology, data collection, data visualization, formal analysis, reviewing, and editing. AK involved in data curation, critical analysis, writing, reviewing, and editing. SD contributed in formal analysis, reviewing, and editing. All authors contributed to the article and approved the submitted version.

## Conflict of Interest

The authors declare that the research was conducted in the absence of any commercial or financial relationships that could be construed as a potential conflict of interest.

## Publisher’s Note

All claims expressed in this article are solely those of the authors and do not necessarily represent those of their affiliated organizations, or those of the publisher, the editors and the reviewers. Any product that may be evaluated in this article, or claim that may be made by its manufacturer, is not guaranteed or endorsed by the publisher.
